# Confronting the “lethal duo” in the ICU: early identification of *Aspergillus–Mucorales* co-infection using a clinical-immuno-inflammatory signature

**DOI:** 10.3389/fcimb.2026.1779186

**Published:** 2026-04-22

**Authors:** Hongqiang Xie, Jiaxu Gu, Junjie Cui, Mengwen Feng, Yonghui Cui, Baoxia Wang, Yi Li, Beiyuan Zhang, Ming Chen

**Affiliations:** 1 Department of Intensive Care Unit, Nanjing Drum Tower Hospital, Clinical College of Xuzhou Medical University, Nanjing, China; 2 Department of Intensive Care Unit, Nanjing Drum Tower Hospital, Nanjing, China; 3 Department of Dermatology, Peking University Shenzhen Hospital, Shenzhen, China; 4 Department of Respiratory and Critical Care Medicine, Nanjing Drum Tower Hospital, Clinical College of Nanjing Medical University, Nanjing, China

**Keywords:** *Aspergillus*, co-infection, intensive care unit, *Mucorales*, prediction model, prognosis

## Abstract

**Background:**

Co-infection with *Aspergillus* and *Mucorales* in the intensive care unit (ICU) represents a devastating syndrome with high mortality that is frequently clinically occult. Clinically distinguishing this co-infection from invasive pulmonary aspergillosis (IPA) is challenging but critical for tailoring precise antifungal strategies.

**Methods:**

We conducted a single-center, retrospective observational study involving 93 critically ill patients (75 with *Aspergillus* infection and 18 with co-infection) admitted between 2017 and 2025. We compared clinical characteristics, inflammatory markers, and immunophenotypes between groups. A three-stage variable selection strategy integrating univariable regression pre-screening, multi-algorithm importance ranking (LASSO, Ridge, and Random Forest), and clinical applicability filtering was employed to identify predictors for a multivariable logistic regression nomogram.

**Results:**

The co-infection group exhibited substantially higher ICU mortality than the sole *Aspergillus* group, although the difference did not reach statistical significance (72.2% vs. 53.3%, *p* = 0.24).Kaplan–Meier analysis demonstrated that initiation of amphotericin B within <7 days of diagnosis or strong clinical suspicion was significantly associated with improved survival (log-rank *p* < 0.0001). A three-stage variable selection strategy integrating univariable regression, multi-algorithm importance ranking (LASSO, Ridge, and Random Forest), and clinical applicability filtering identified four key predictors. The resulting multivariable logistic regression nomogram — incorporating NK cell count, C-reactive protein, corticosteroid use history, and Gram-positive bacterial co-infection — demonstrated robust discrimination (AUC = 0.878, 95% CI: 0.789–0.967), with good calibration (Hosmer–Lemeshow *p* = 0.849) and stability on internal validation (cross-validated AUC = 0.860).

**Conclusion:**

*Aspergillus* and *Mucorales* co-infection constitutes a distinct, high-mortality clinical entity in the ICU. The developed nomogram, integrating clinical, immunological, and inflammatory features, may facilitate the early identification of high-risk patients and guide timely initiation of *Mucorales*-active therapy to improve prognosis.

## 1 Introduction

Invasive pulmonary aspergillosis (IPA) and pulmonary mucormycosis (PM) constitute the two predominant etiologies of invasive mold infections (IMIs) in the intensive care unit (ICU). While IPA represents the most common entity, its mortality rate in critically ill patients typically ranges from 40% to 50% despite standard frontline therapy (Taccone et al., 2015). Conversely, PM has historically been burdened with a significantly graver prognosis. Although recent meta-analyses suggest that advancements in early intervention have reduced the mortality of pure PM to approximately 50% to 60% (Muthu et al., 2021), it remains a devastating condition. Against this backdrop, the emergence of mixed *Aspergillus* and *Mucorales* co-infections presents a compounded clinical threat. Epidemiological data indicate that 10%–20% of patients with confirmed mucormycosis harbor concurrent aspergillosis (Ra et al., 2024). This “lethal duo” poses an extreme diagnostic challenge: unlike pure IPA, co-infections are frequently misdiagnosed as solitary aspergillosis, leading to the empirical use of voriconazole—an agent intrinsically ineffective against *Mucorales*. Consequently, driven by diagnostic delays and inappropriate initial therapy, the mortality rate for co-infections remains alarmingly high, reported to reach 70% to 88% (Sasani et al., 2024; Ra et al., 2024), significantly exceeding that of pure *Aspergillus* infections and rivaling the poorest outcomes of disseminated *mucormycosis*.

Given the intrinsic resistance of *Mucorales* species to voriconazole—the frontline therapeutic agent for aspergillosis—the early identification of co-infection and the prompt initiation of broad-spectrum antifungal regimens capable of covering both pathogens (e.g., liposomal amphotericin B) are paramount for improving patient outcomes (Lewis and Kontoyiannis, 2013). However, the early differential diagnosis of co-infection poses significant clinical challenges. Radiologically, pathognomonic features of mucormycosis, such as the reverse halo sign, are frequently obscured by the imaging manifestations of aspergillosis, resulting in a lack of diagnostic specificity (Legouge et al., 2014). Microbiologically, the sensitivity of traditional fungal culture for *Mucorales* remains notoriously low (approximately 30%–40%) (Lackner et al., 2021; Skiada et al., 2020), leading to frequent diagnostic oversight. Consequently, if clinicians rely solely on positive evidence of *Aspergillus* to initiate azole monotherapy, they risk a catastrophic clinical scenario: such treatment not only fails to contain *Mucorales* but may arguably accelerate the progression of occult mucormycosis through selective pressure, precipitating inevitable treatment failure (Lamaris et al., 2009).

Despite extensive research delineating the individual characteristics of sole IPA or PM, there remains a paucity of direct comparative studies focusing on the distinction between “sole *Aspergillus* infection” and “mixed *Aspergillus*-*Mucorales* co-infection” (Jeong et al., 2019). Consequently, the specific clinical and immunological landscape of this mixed entity remains largely uncharted, leaving clinical practice without effective early prediction tools for risk stratification. To bridge this critical knowledge gap, the present study aims to systematically characterize the distinctive immunophenotypes, inflammatory profiles, and clinical comorbidities of co-infection versus sole *Aspergillus* infection through a retrospective analysis of a critically ill cohort. Furthermore, we sought to construct and validate a multidimensional early risk prediction model to assist clinicians in the rapid identification of high-risk populations, thereby guiding the timely adjustment of antifungal strategies to optimize therapeutic decision-making and ultimately improve survival outcomes.

## 2 Materials and methods

### 2.1 Study design and participants

This single-center, retrospective, observational cohort study was conducted using the clinical database of the Department of Critical Care Medicine at Nanjing Drum Tower Hospital, The Affiliated Hospital of Nanjing University Medical School, Nanjing, China. The study protocol adhered to the ethical principles of the Declaration of Helsinki and was approved by the Institutional Ethics Committee (Approval No. 2023-602). Due to the retrospective nature of the study and the de-identification of patient data, the requirement for informed consent was waived.

**Figure 1:**
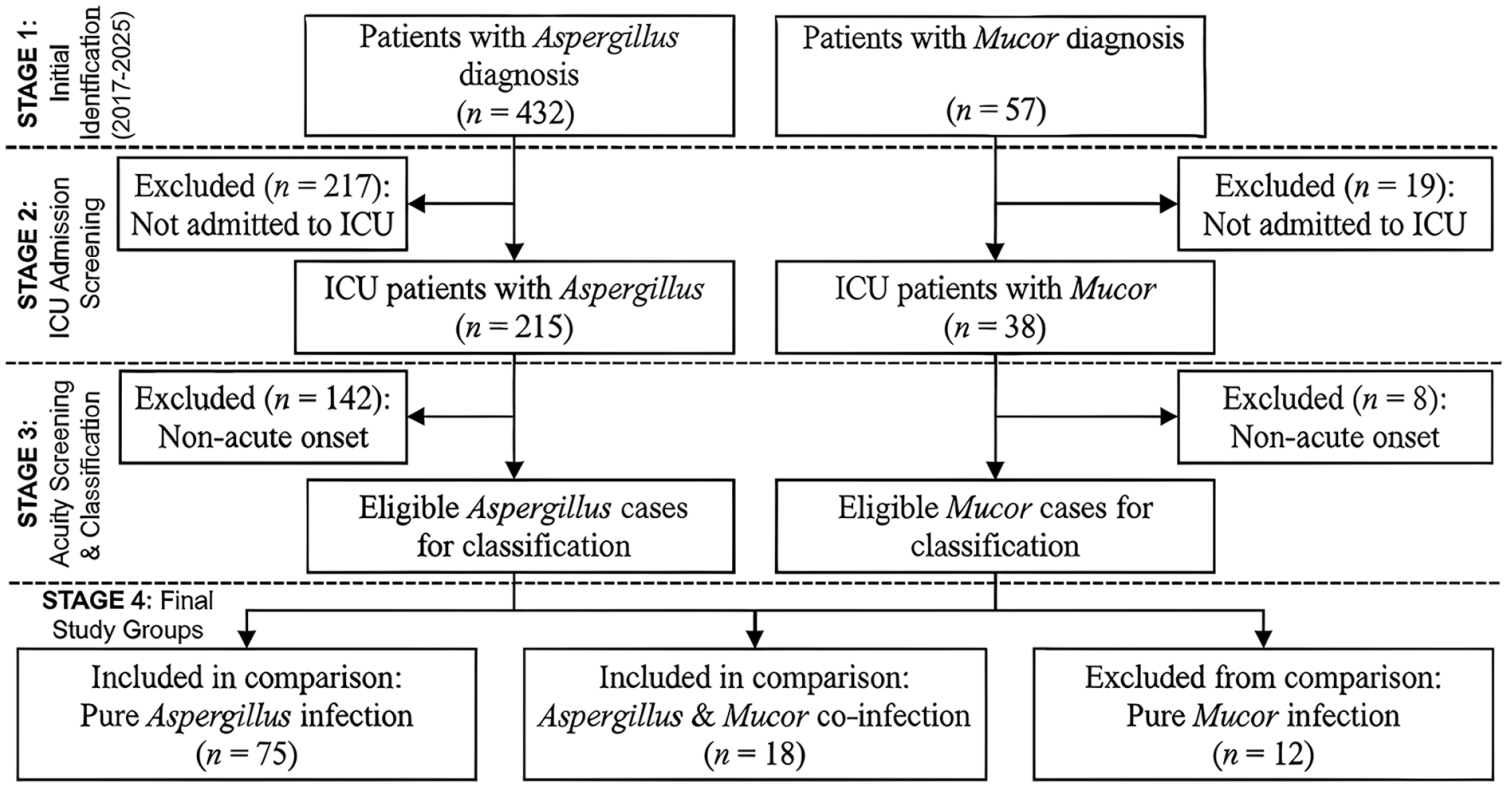
Flowchart of patient recruitment and study group stratification. Initial screening of the ICU clinical database (2017–2025) identified a primary pool of patients with *Aspergillus* (n = 432) or *Mucorales* (n = 57) infections. Following the sequential application of exclusion criteria (e.g., non-ICU admission, absence of acute onset), eligible patients were stratified into two comparative cohorts: the Sole *Aspergillus* Infection Group (n = 75) and the Mixed *Aspergillus* and *Mucorales* Co-infection Group (n = 18). ICU, Intensive Care Unit.

Electronic medical records were systematically reviewed from January 1, 2017, to December 31, 2025. Following a predefined screening algorithm (**Figure 1**), we initially identified 432 patients with a primary diagnosis of *Aspergillus* infection and 57 patients with a primary diagnosis of mucormycosis. To ensure cohort homogeneity, we sequentially excluded cases that were not admitted to the ICU or did not present with an acute onset. Subsequently, the eligible cohort was stratified based on co-infection status into two distinct groups: the Sole *Aspergillus* Infection Group (*n* = 75), defined as patients with evidence of *Aspergillus* infection in the absence of *Mucorales* co-infection; and the Co-infection Group (*n* = 18), defined as patients demonstrating microbiological, molecular, or pathological evidence of simultaneous infection with both *Aspergillus* and *Mucorales*. Cases of sole mucormycosis (*n* = 12) were excluded from this comparative analysis to focus specifically on distinguishing co-infection from monomicrobial aspergillosis.

### 2.2 Diagnostic criteria

Diagnoses of invasive mold infection (IMI) were classified according to the revised consensus definitions of the European Organization for Research and Treatment of Cancer and the Mycoses Study Group Education and Research Consortium (EORTC/MSGERC) (Donnelly et al., 2020), with adaptive modifications to accommodate the clinical constraints of the ICU setting. Invasive Pulmonary Aspergillosis (IPA): Cases were categorized as either proven or probable. A proven diagnosis necessitated histopathological confirmation from lung tissue or a positive culture from a sterile site. A probable diagnosis was defined by the concurrent presence of host factors, compatible pulmonary radiological features, and mycological evidence. Mycological criteria included the isolation of *Aspergillus* spp. from lower respiratory tract specimens (bronchoalveolar lavage fluid [BALF] or qualified sputum) or a positive galactomannan (GM) assay in BALF (optical density index≥0.8). Pulmonary Mucormycosis (PM): A proven diagnosis was based on the histopathological demonstration of characteristic broad, pauciseptate hyphae with right-angle branching. For critically ill patients unable to tolerate invasive biopsy, a probable diagnosis (clinical diagnosis) required the simultaneous presence of a high-risk background, imaging findings indicative of pulmonary necrosis (e.g., reversed halo sign, cavitation), and at least one of the following microbiological criteria: (1) isolation of *Mucorales* from sputum or BALF culture; or (2) identification of *Mucorales*-specific DNA sequences in body fluids via metagenomic next-generation sequencing (mNGS). All mNGS testing was performed by a nationally certified clinical laboratory, with results interpreted in accordance with the 2021 Chinese Expert Consensus on mNGS in Infectious Disease Diagnosis. Given the absence of a universally accepted sequence read-count threshold and the inherent difficulties in sampling and cell lysis for *Mucorales*, low read-count detections were considered clinically meaningful only after bioinformatic confirmation, cross-validation with alternative methodologies, and comparison against same-run negative controls. The final distinction between true infection and colonisation was adjudicated by a multidisciplinary team (MDT) comprising infectious disease specialists, intensivists, and clinical microbiologists, based on integrated review of host risk factors, clinical manifestations, radiological findings, and microbiological results.

### 2.3 Statistical analysis

Statistical analyses were performed using SPSS (version 31.0), R (version 4.5.2), GraphPad Prism (version 10), and Python (version 3.14). Continuous variables were compared using Student’s *t*-test or the Mann–Whitney U test, as appropriate; categorical variables were analysed using the Chi-square or Fisher’s exact test. A two-sided *p* < 0.05 was considered statistically significant.To identify predictors of *Aspergillus*–*Mucorales* co-infection, a three-stage variable selection strategy was employed (**Supplementary Figure S1**): univariable logistic regression screening, multi-algorithm importance ranking (LASSO, Ridge, and Random Forest), and clinical applicability filtering. Variables with consistently high algorithmic importance were entered into a multivariable logistic regression model. Model performance was assessed by discrimination (AUC with 95% CI, DeLong method), calibration (bootstrap resampling, B = 1,000), and internal validation (10-repetition 5-fold cross-validation). Survival was estimated by the Kaplan–Meier method with log-rank testing; time zero was defined as the date of diagnosis or strong clinical suspicion and initiation of antifungal therapy.

## 3 Results

### 3.1 Baseline characteristics

A total of 93 critically ill patients meeting the inclusion criteria were enrolled and categorized into the Sole *Aspergillus* Infection Group (n = 75) and the Co-infection Group (n = 18). Demographic baselines (age, gender) and major comorbidities—including hypertension, diabetes mellitus, malignancy, and solid organ transplantation—were well-balanced between the two cohorts, with no statistically significant differences observed (**Table 1**). Notably, the prevalence of antecedent corticosteroid exposure was significantly higher in the sole *Aspergillus* group than in the co-infection group (64.0% vs. 27.8%, *p* = 0.01). Corticosteroid exposure patterns are detailed in the footnotes of **Table 1**.

**Table 1:** Baseline characteristics, clinical features, and laboratory findings of patients with *Aspergillus* infection and *Aspergillus*-*Mucor* co-infection.

Variable	Overall (n = 93)	*Aspergillus* (n = 75)	Co-infection (n = 18)	p value
Demographics				
Male sex, n (%)	54 (58.1%)	43 (57.3%)	11 (61.1%)	0.98
Age, years	59 (50, 71)	61 (50.5, 72)	57.5 (50.5, 63.5)	0.25
Symptom onset to admission, days	5.00 (1.75, 7.00)	6.00 (1.25, 7.00)	3.50 (2.00, 6.50)	0.318
Symptom onset to diagnosis, days	11.5 (7.75, 18.3)	11.0 (7.25, 17.0)	15.0 (9.00, 20.5)	0.312
Length of hospital stay, days	26.0 (15.8, 50.0)	27.5 (15.0, 50.0)	23.0 (18.3, 46.3)	0.852
SOFA score	9.00 (6.00, 12.0)	9.00 (6.00, 11.0)	10.00 (8.50, 12.8)	0.174
APACHE II score	20.92 ± 8.40	20.83 ± 8.34	21.25 ± 8.90	0.861
ICU Mortality, n (%)	53 (57%)	40 (53.3%)	13 (72.2%)	0.24
Comorbidities, n (%)				
Hypertension	38 (40.9%)	31 (41.3%)	7 (38.9%)	1
Coronary heart disease	10 (10.8%)	8 (10.7%)	2 (11.1%)	1
Diabetes mellitus	24 (25.8%)	18 (24%)	6 (33.3%)	0.61
COPD	31 (33.3%)	27 (36.0%)	4 (22.2%)	0.40
Liver cirrhosis	11 (11.8%)	10 (13.3%)	1 (5.6%)	0.69
Malignancy	11 (11.8%)	7 (9.3%)	4 (22.2%)	0.21
Solid organ transplantation	6 (6.5%)	5 (6.7%)	1 (5.6%)	1
Autoimmune disease	19 (20.4%)	16 (21.3%)	3 (16.7%)	1
Corticosteroid use	53 (57%)	48 (64%)	5 (27.8%)	0.01
Long-term oral/inhaled	40 (43.0%)	37 (49.3%)	3 (16.7%)	
Short-term intravenous pulse	13 (14.0%)	11 (14.7%)	2 (11.1%)	
Complications & Coinfections, n (%)				
Respiratory failure	82 (88.2%)	64 (85.3%)	18 (100%)	0.12
Septic shock	62 (66.7%)	48 (64%)	14 (77.8%)	0.4
Acute kidney injury	63 (67.7%)	50 (66.7%)	13 (72.2%)	0.86
Gastrointestinal dysfunction	34 (36.6%)	25 (33.3%)	9 (50%)	0.3
Liver dysfunction	66 (71%)	51 (68%)	15 (83.3%)	0.26
Gram-positive bacteria	60 (64.5%)	44 (58.7%)	16 (88.9%)	0.03
Gram-negative bacteria	76 (81.7%)	60 (80%)	16 (88.9%)	0.51
Viral coinfection	64 (68.8%)	52 (69.3%)	12 (66.7%)	1
Extrapulmonary infection	3 (3.2%)	0 (0%)	3 (16.7%)	0.01
ICU Interventions, n (%)				
Mechanical ventilation	75 (80.6%)	58 (77.3%)	17 (94.4%)	0.18
CRRT	60 (64.5%)	48 (64%)	12 (66.7%)	1
ECMO	9 (9.7%)	5 (6.7%)	4 (22.2%)	0.07
Laboratory Findings				
White blood cell count, ×10^9^/L	9.00 (5.00, 15.2)	9.00 (5.45, 13.9)	9.90 (4.17, 16.3)	0.785
Neutrophil count, ×10^9^/L	6.70 (3.80, 14.2)	6.50 (3.80, 12.4)	8.90 (3.77, 16.7)	0.380
C-reactive protein, mg/L	93.1 (24.2, 152.4)	77.7 (19.2, 126.8)	175.6 (99.0, 246.4)	0.001
Procalcitonin, ng/mL	0.78 (0.29, 3.95)	0.65 (0.25, 2.83)	1.85 (0.91, 16.39)	0.069
Total protein, g/L	53.63 ± 9.25	53.80 ± 9.77	52.88 ± 6.67	0.712
Albumin, g/L	30.27 ± 4.91	30.33 ± 5.24	29.99 ± 3.06	0.801
Interleukin-6, pg/mL	127.1 (33.9, 493.9)	94.0 (32.1, 333.1)	340.0 (73.0, 1342.5)	0.084
Platelet count, ×10^9^/L	94.0 (33.0, 164.0)	89.0 (33.5, 165.5)	106.5 (37.3, 163.8)	0.969
RBC, ×10^12^/L	3.35 (2.73, 4.21)	3.44 (2.87, 4.35)	2.88 (2.56, 3.69)	0.073
Hemoglobin, g/L	103.2 ± 29.4	106.3 ± 28.2	90.6 ± 31.6	0.041
Fibrinogen, g/L	3.40 (2.20, 4.90)	3.20 (2.10, 4.85)	4.40 (3.42, 5.42)	0.033
D-dimer, mg/L	3.42 (1.96, 7.96)	3.31 (1.82, 6.54)	3.90 (2.67, 11.9)	0.304
Total Lymphocytes, ×10^9^/L	0.7 (0.3, 1)	0.70 (0.37, 1.10)	0.30 (0.15, 0.60)	0.006
B cells, ×10^9^/L	0.10 (0.05, 0.23)	0.10 (0.05, 0.30)	0.10 (0.03, 0.13)	0.165
CD3+CD4+ T cells, ×10^9^/L	0.15 (0.08, 0.30)	0.17 (0.10, 0.34)	0.05 (0.02, 0.19)	0.003
CD3+CD8+ T cells, ×10^9^/L	0.16 (0.08, 0.30)	0.18 (0.10, 0.32)	0.10 (0.05, 0.16)	0.034
CD3+ T cells, ×10^9^/L	0.35 (0.18, 0.60)	0.42 (0.20, 0.61)	0.20 (0.08, 0.41)	0.023
NK cells, ×10^9^/L	0.05 (0.03, 0.19)	0.09 (0.03, 0.20)	0.03 (0.02, 0.04)	0.003
Creatinine, μmol/L	87.1 (55.0, 173.9)	81.0 (55.0, 164.4)	92.0 (57.0, 201.0)	0.744
eGFR, mL/min/1.73m^2^	79.1 (33.2, 128.6)	80.6 (33.9, 127.7)	66.8 (30.1, 129.0)	0.994
Total bilirubin, μmol/L	16.1 (10.3, 50.5)	15.9 (9.40, 51.9)	23.1 (12.5, 30.1)	0.497
Direct bilirubin, μmol/L	6.3 (3.2, 25.1)	6.2 (3.15, 26.95)	8.55 (4.6, 18.75)	0.91
Galactomannan (GM) test, ODI	0.53 (0.21, 2.19)	0.49 (0.25, 2.26)	0.94 (0.15, 2.08)	0.973
(1,3)-β-D-glucan (BDG), pg/mL	101.5 (10.0, 241.2)	139.7 (10.0, 267.1)	49.98 (10.0, 102.6)	0.082
CT Findings, n (%)				
Tracheal stenosis/occlusion	9(9.7%)	6 (8.0%)	3 (16.7%)	0.38
Wedge-shaped consolidation	24(25.8%)	18 (24.0%)	6 (33.3%)	0.42
Multiple nodules	53(60.0%)	42 (56.0%)	11 (61.1%)	0.78
Cavitation	26(28.0%)	20 (26.7%)	6 (33.3%)	0.57
Reverse halo sign	5(5.4%)	3 (4.0%)	2 (11.1%)	0.25

APACHE II, Acute Physiology and Chronic Health Evaluation II; BDG, (1,3)-beta-D-glucan; CD, Cluster of Differentiation; CHD, Coronary Heart Disease; CRP, C-reactive Protein; CRRT, Continuous Renal Replacement Therapy; CT, Computed Tomography; ECMO, Extracorporeal Membrane Oxygenation; COPD, chronic obstructive pulmonary disease;eGFR, estimated Glomerular Filtration Rate; GM, Galactomannan; ICU, Intensive Care Unit; IL-6, Interleukin-6; IQR, Interquartile Range; NK cells, Natural Killer cells; ODI, Optical Density Index; PCT, Procalcitonin; SD, Standard Deviation; SOFA, Sequential Organ Failure Assessment. Long-term oral/inhaled corticosteroid use was defined as a prednisone-equivalent dose of ≥ 0.3 mg/kg/day for > 3 weeks, in accordance with the EORTC/MSGERC host factor criteria. Short-term intravenous pulse therapy was defined as methylprednisolone ≥ 250 mg/day for 3–5 consecutive days.

### 3.2 Clinical features and co-infection patterns

The analysis revealed that a distinguishing characteristic of the co-infection cohort was the high burden of bacterial superinfection. Specifically, the prevalence of concurrent Gram-positive bacterial infection was significantly elevated in the Co-infection Group compared to the Sole *Aspergillus* Infection Group (88.9% vs. 58.7%, *p* = 0.03).

Regarding tissue invasiveness, the Co-infection Group exhibited a marked propensity for extrapulmonary dissemination. While pulmonary involvement was universal in both cohorts, disseminated disease involving the sino-orbital region, central nervous system (CNS), and cutaneous/soft tissues was confirmed in 3 patients (16.7%) within the mixed group. In stark contrast, no such dissemination was observed in the Sole *Aspergillus* Group (*p* = 0.01). In terms of organ support, there was a notable trend toward increased utilization of extracorporeal membrane oxygenation (ECMO) in the mixed group (22.2% vs. 6.7%). Although this difference did not reach statistical significance (*p* = 0.07), it suggests a potentially more severe trajectory of respiratory failure in this population.

### 3.3 Diagnostic methodology analysis

Diagnostic validation relied on a composite of clinical, microbiological, and pathological evidence. In the Sole *Aspergillus* Group (*n* = 75), microbiological confirmation was primarily derived from positive lower respiratory tract cultures (47 cases, 62.7%) and mNGS (61 cases, 81.3%). Conversely, the Co-infection Group (*n* = 18) underscored the inherent limitations of conventional diagnostics: *Mucorales* species were isolated via culture in only 3 patients (16.7%), a finding consistent with the fragile, fastidious growth requirements and historically low sensitivity of culture for these fungi (Lamaris et al., 2009; Jeong et al., 2019). In contrast, mNGS technology demonstrated superior diagnostic utility, identifying *Mucorales*-specific sequences in 16 patients (88.9%) and effectively bridging the diagnostic gap. Additionally, definitive histopathological diagnosis—characterized by broad, pauciseptate hyphae with right-angle branching—was obtained via tissue biopsy in 2 patients (11.1%).

### 3.4 Immunological and inflammatory profile

Laboratory investigations elucidated a distinct phenotype characterized by severe inflammatory dysregulation and immune suppression in the Co-infection Group (**Table 1**). Regarding systemic inflammation, the co-infection cohort exhibited a markedly elevated burden, with C-reactive protein (CRP) levels significantly exceeding those of the Sole *Aspergillus* Infection Group (median 175.6 vs. 77.7 mg/L, *p* = 0.001). Concomitant with this hyperinflammatory state, hemoglobin levels were significantly depressed in the mixed group (90.6 ± 31.6 vs. 106.3 ± 28.2 g/L, *p* = 0.041), suggestive of severe infection-associated anemia or a heightened catabolic state.

The most striking disparities emerged from the immunophenotyping analysis. Compared to the Sole *Aspergillus* Infection Group, the Co-infection Group demonstrated a “dual impairment” involving both innate and adaptive immunity. Specifically, absolute counts of Natural Killer (NK) cells were significantly suppressed (*p* = 0.003). Simultaneously, CD3+CD8+ cytotoxic T lymphocytes (CTLs) exhibited significant depletion (0.10 vs. 0.18, *p* = 0.034). Collectively, these findings indicate that patients with co-infection are in a state of profound “immunoparesis”, characterized by the loss of critical cytotoxic clearance capabilities.

### 3.5 Construction of the prediction model

**Figure 2:**
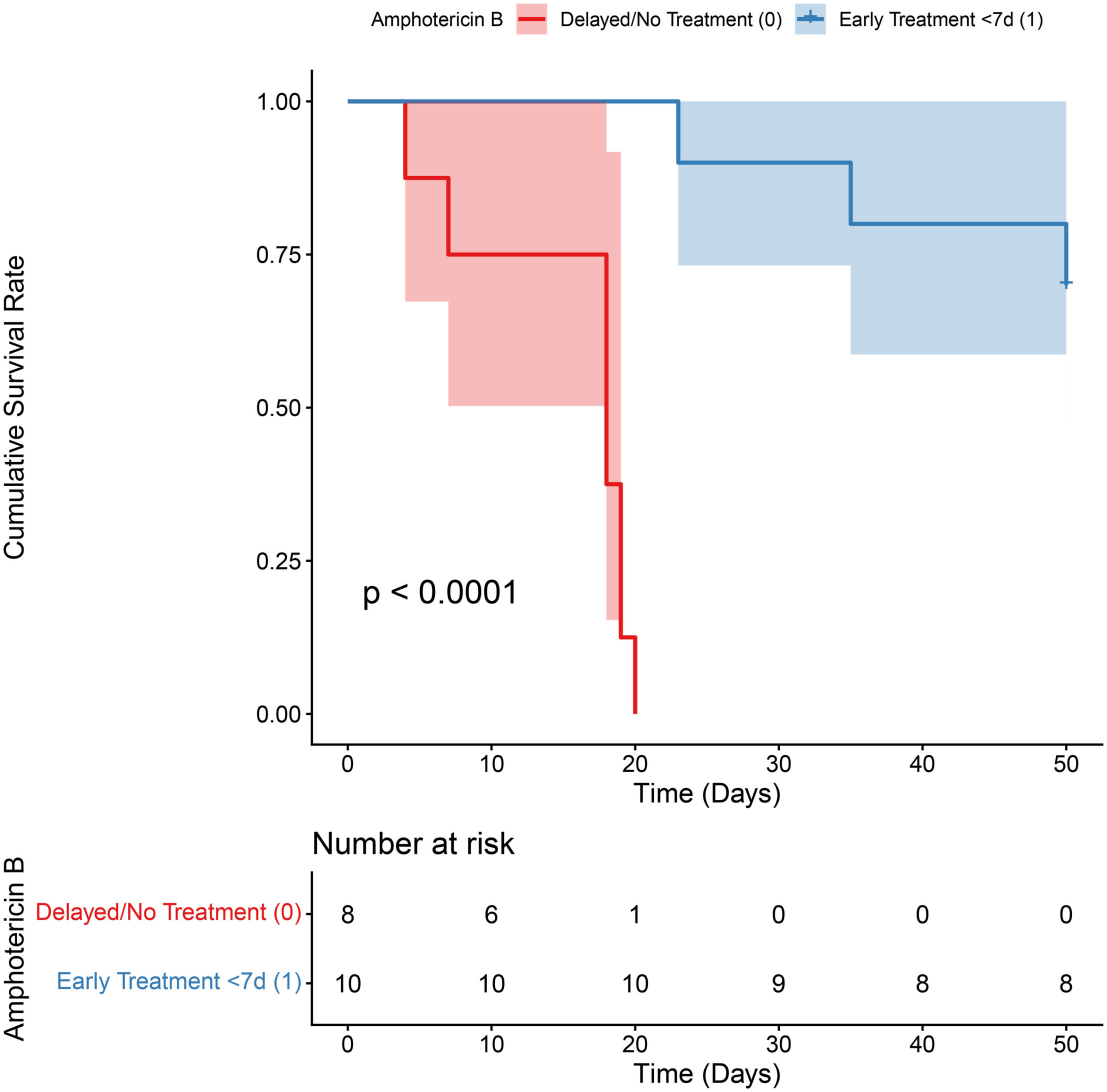
Kaplan-Meier survival estimates stratified by the timing of Mucor-active antifungal therapy initiation. The blue line represents patients who initiated amphotericin B therapy within the critical therapeutic window (<7 days from the date of diagnosis or strong clinical suspicion of co-infection, defined as time zero), while the red line represents patients with delayed or absent *Mucorales*-active therapy. Treatment timing was analysed as a fixed baseline exposure. Shaded areas indicate the 95% confidence intervals. The difference in cumulative survival rates between the two groups was statistically significant (log-rank test, *p* < 0.0001).

**Figure 3:**
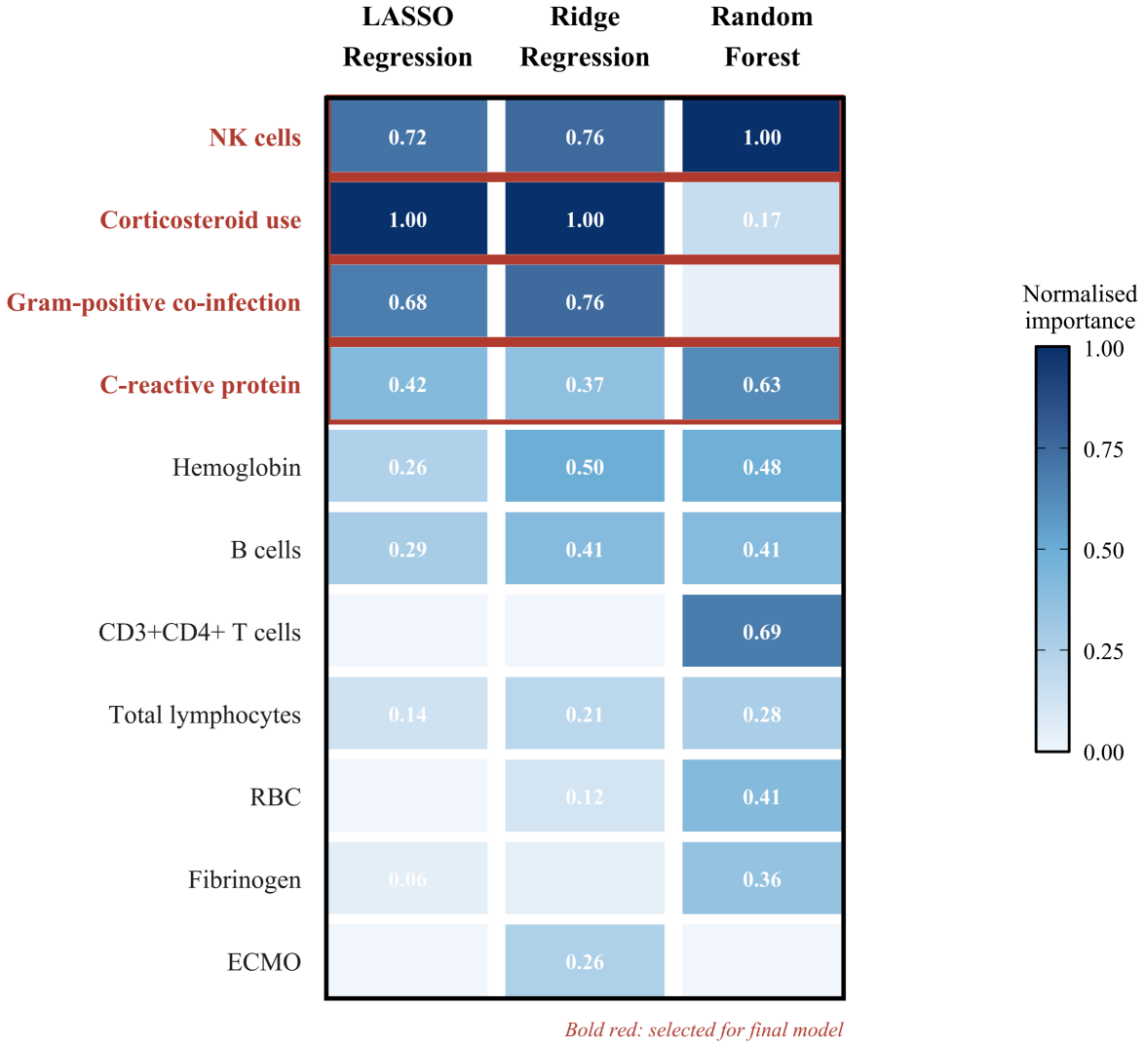
Heatmap of feature importance rankings across three machine learning algorithms for the 11 candidate variables. The heatmap displays normalised importance scores (0–1) of 11 candidate variables (univariable p < 0.1) across LASSO, Ridge, and Random Forest algorithms. Darker shading indicates higher importance. The four variables in bold red—NK cell count, corticosteroid use, Gram-positive bacterial co-infection, and CRP—were selected for the final multivariable model. Abbreviations: LASSO, least absolute shrinkage and selection operator; OOB, out-of-bag.

**Figure 4:**
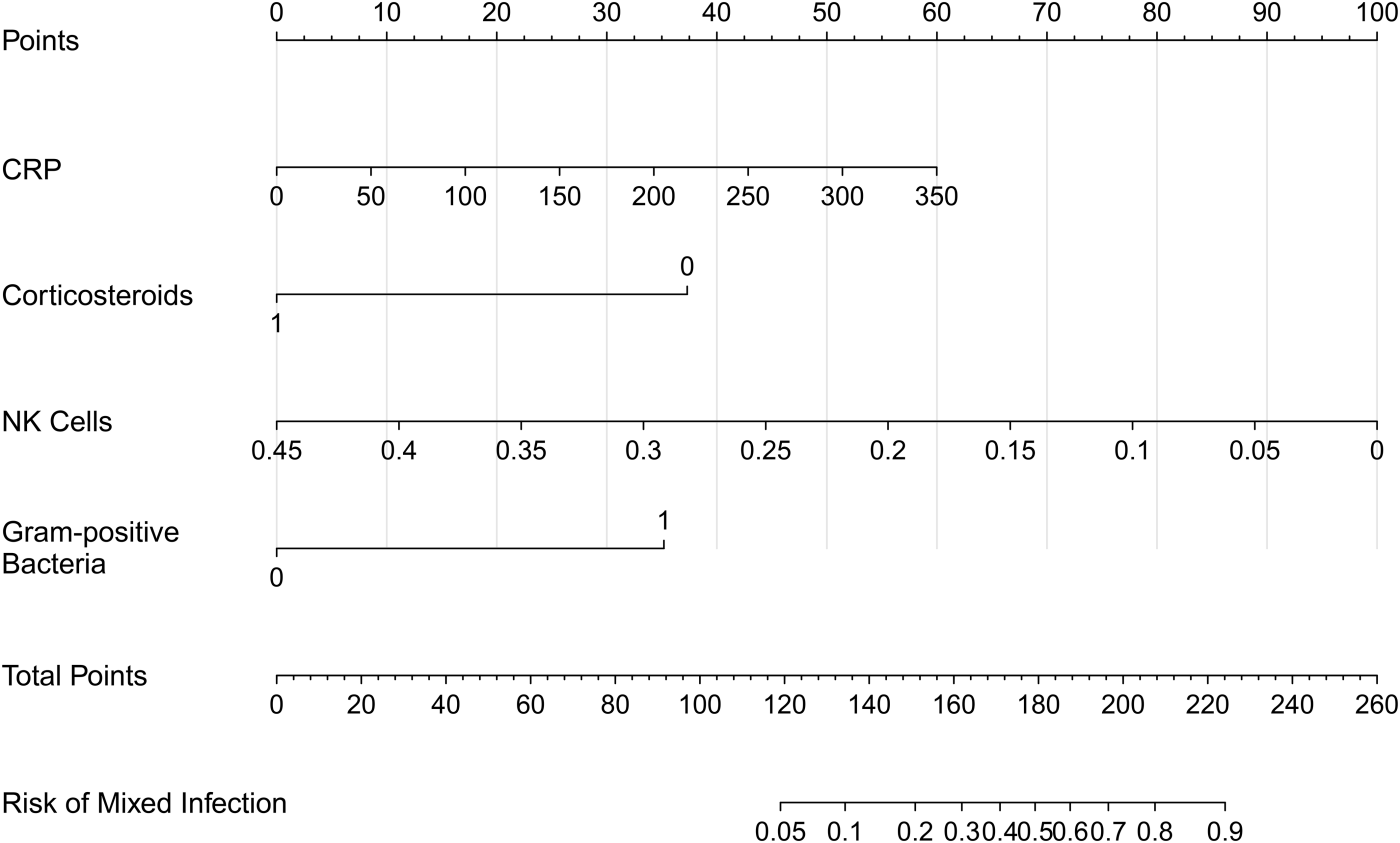
Diagnostic nomogram for predicting the probability of mixed *Aspergillus* and *Mucorales* co-infection. The nomogram allows for the calculation of individual risk based on the multivariate model. Instructions for use: Locate the patient’s specific value on each variable axis. Project a vertical line upward to the “Points” scale to determine the score assigned to each predictor. Sum the scores from all four variables to obtain the “Total Points.” Finally, locate this aggregate score on the “Total Points” axis and project a vertical line downward to the “Risk” axis to read the predicted probability of co-infection. Abbreviations: NK, Natural Killer; G+, Gram-positive bacteria; CRP, C-reactive protein.

**Figure 5:**
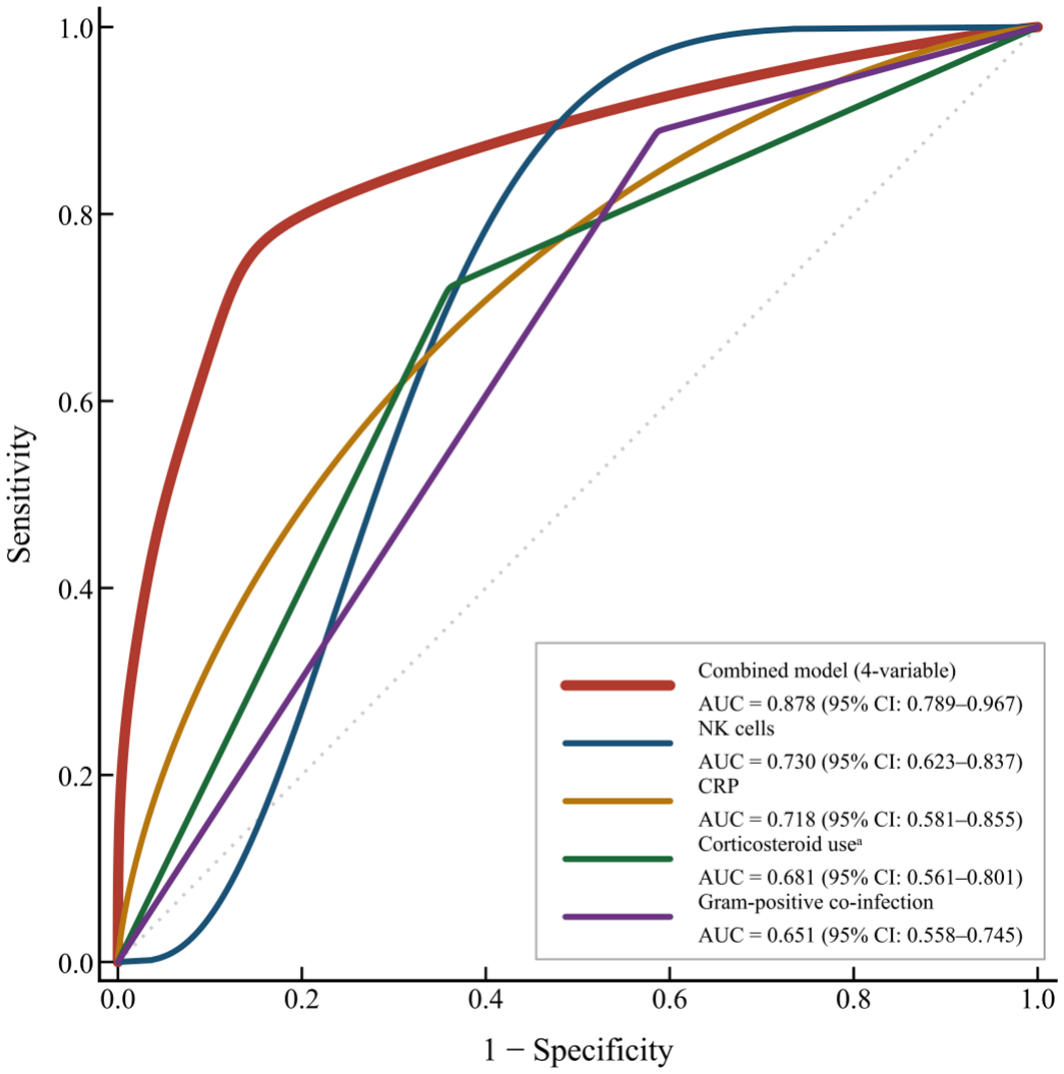
Receiver operating characteristic (ROC) curves comparing the discriminatory power of the multivariate nomogram against individual predictors. The composite model (bold red line), integrating NK cell count, CRP, corticosteroid history, and Gram-positive bacterial co-infection, demonstrated superior predictive performance with an AUC of 0.878 (95% CI: 0.789–0.967). This value notably exceeded the discriminative ability of individual variables, including NK cells (AUC = 0.730, 95% CI: 0.623–0.837), CRP (AUC = 0.718, 95% CI: 0.581–0.855), corticosteroid use (AUC = 0.681, 95% CI: 0.561–0.801), and Gram-positive bacterial co-infection (AUC = 0.651, 95% CI: 0.558–0.745). AUC values and 95% CIs were estimated using the DeLong method. The diagonal grey line represents the reference line of no discrimination (AUC = 0.5). ^a^ For corticosteroid use, the ROC curve was plotted in the direction of absence of corticosteroid exposure as the positive predictor, reflecting its role as a negative independent predictor in the multivariable model (OR = 0.161).

**Figure 6:**
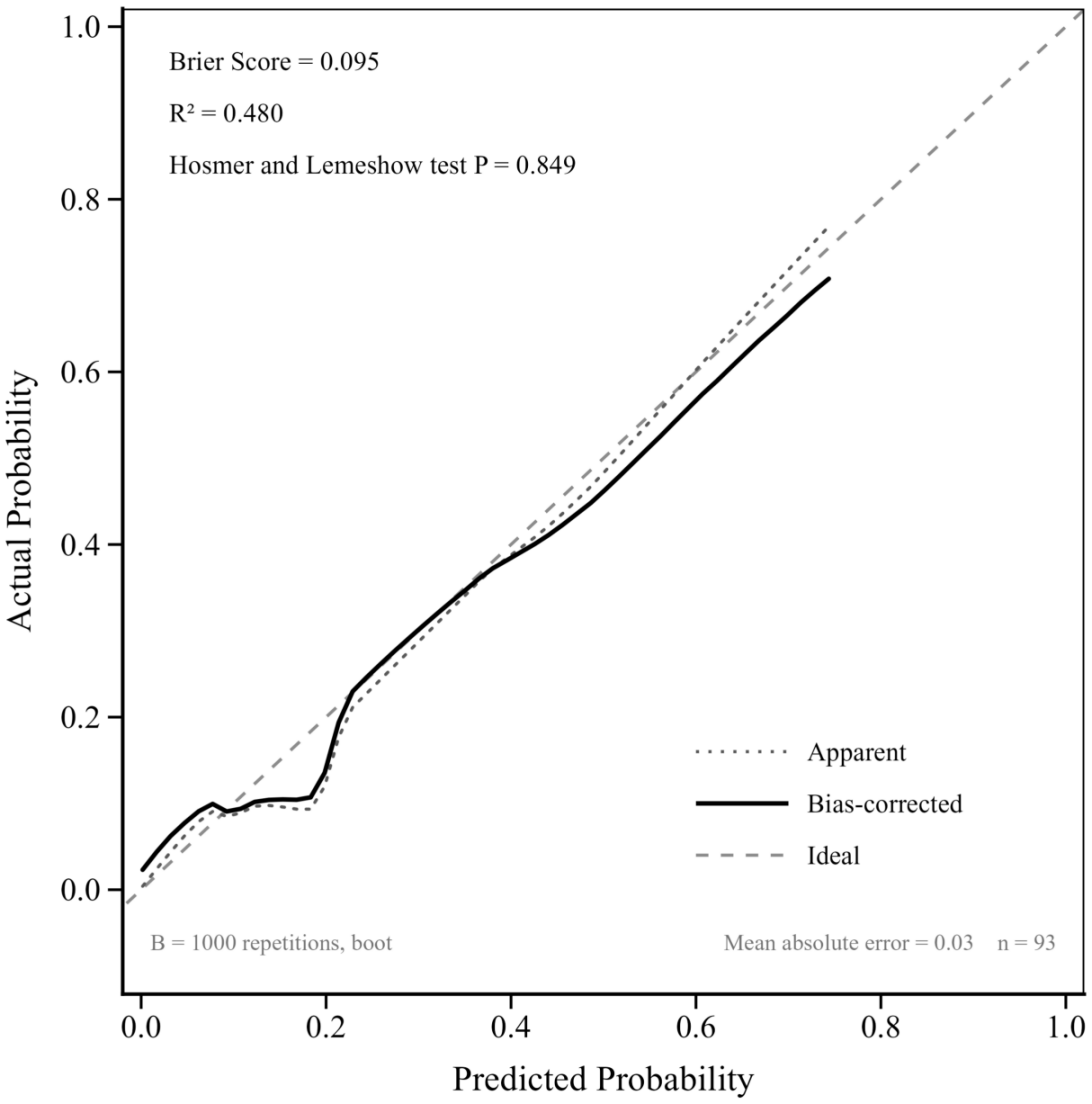
Calibration curve assessing the goodness-of-fit for the predictive nomogram. The calibration curve evaluates agreement between predicted and observed probabilities. The solid line indicates bias-corrected performance via Bootstrap resampling (B = 1,000), and the dotted line represents apparent accuracy. The model demonstrated excellent calibration (Brier score = 0.095; Nagelkerke R^2^ = 0.480; Hosmer–Lemeshow p = 0.849; bias-corrected mean absolute error = 0.026; n = 93).

Kaplan–Meier analysis demonstrated that initiation of amphotericin B within a 7-day therapeutic window from time zero was significantly associated with improved survival in patients with *Aspergillus*–*Mucorales* co-infection (log-rank *p* < 0.0001; **Figure 2**). Univariable screening identified 11 candidate variables (*p* < 0.1; **Supplementary Table S1**), which were subsequently ranked by LASSO, Ridge, and Random Forest algorithms (**Figure 3**). Four variables demonstrating consistently high importance were selected for multivariable logistic regression (**Table 2**) and visualised as a diagnostic nomogram (**Figure 4**): NK cell count, CRP, corticosteroid use history, and Gram-positive bacterial co-infection. The combined model achieved an AUC of 0.878 (95% CI: 0.789–0.967; **Figure 5**), substantially exceeding that of any individual predictor. Calibration was excellent (Brier score = 0.095; Hosmer–Lemeshow *p* = 0.849; **Figure 6**), and 10-repetition 5-fold cross-validation confirmed negligible overfitting (mean AUC = 0.860, optimism = 0.017).

**Table 2:** Multivariable logistic regression analysis of predictors for mixed *Aspergillus–Mucorales* co-infection.

Variable	β	OR	95% CI	p value
NK cell count (×10^9^/L)	-10.876	0.000	0.000 – 0.061	0.039
C-reactive protein (mg/L)	0.008	1.008	1.001 – 1.016	0.024
Gram-positive bacterial co-infection	1.722	5.597	1.110 – 46.533	0.061
Corticosteroid use	-1.826	0.161	0.038 – 0.581	0.008

Model fit: Nagelkerke R^2^ = 0.480; Hosmer–Lemeshow goodness-of-fit test *p* = 0.849. n = 93; outcome events (co-infection) = 18; events-per-variable ratio = 4.5. Abbreviations: β, regression coefficient; OR, odds ratio; CI, confidence interval. Bold *p* values indicate statistical significance (*p* < 0.05). Gram-positive bacterial co-infection did not reach conventional significance but was retained based on consistent high-importance ranking across all three machine learning algorithms and established clinical relevance (univariable OR = 5.636, *p* = 0.028).

## 4 Discussion

In the present study, we systematically compared the clinical and immuno-inflammatory profiles of *Aspergillus*–*Mucorales* co-infection versus sole IPA in critically ill patients. Compared with sole IPA, patients with co-infection exhibited a distinct phenotype characterised by more severe immune suppression (depleted NK cells and CD8+ T-cell subsets), heightened systemic inflammation (elevated CRP), and a higher burden of Gram-positive bacterial co-infection. Building on these observations, a multivariable logistic regression model incorporating four clinically accessible variables — NK cell count, CRP, corticosteroid use history, and Gram-positive bacterial co-infection — demonstrated robust discrimination (AUC = 0.878, 95% CI: 0.789–0.967), providing a potential tool for early identification of co-infection in the ICU setting.

A pivotal finding of our study is that patients with *Aspergillus*–*Mucorales* co-infection exhibited a state of profound immunoparesis that was significantly distinct from sole *Aspergillus* infection. Our model identified reduced NK cell count as one of the strongest independent predictors for distinguishing co-infection. Previous studies have confirmed that NK cells, as critical effectors of antifungal innate immunity, directly kill fungal hyphae through the release of perforin and granzymes (Ogbomo and Mody, 2017-; Schmidt et al., 2017), and their depletion is closely associated with the dissemination of invasive mold infections (Park et al., 2009). Additionally, our data demonstrated significant depletion of CD3+CD8+ T cells (*p* = 0.034) in the co-infection group, suggesting concurrent impairment of both innate and adaptive antifungal immunity (Hotchkiss et al., 2013). This immunoparetic state may be perpetuated by the high burden of bacterial co-infection observed in the co-infection group, which was notably pronounced for both Gram-positive (88.9% vs. 58.7%, *p* = 0.03) and Gram-negative pathogens (88.9% vs. 80.0%, *p* = 0.51), as bacterial sepsis is known to drive widespread lymphocyte apoptosis and T-cell exhaustion (Lionakis and Kontoyiannis, 2003; Wang et al., 2021). Consequently, the host loses critical immune clearance capacity against the aggressive angioinvasion characteristic of *Mucorales*, ultimately contributing to poor prognosis (Baldin and Ibrahim, 2017).

Contrary to traditional paradigms where corticosteroids are viewed as universal risk factors for invasive fungal disease (IFD) (Lionakis and Kontoyiannis, 2003; Kousha et al., 2011), our model identified antecedent corticosteroid usage as a negative predictor for co-infection. This finding should not be misconstrued as corticosteroids conferring a protective effect against mucormycosis. Rather, it reflects distinct host heterogeneities between the two cohorts. Sole IPA typically occurs in the context of chronic immunosuppression, predominantly in patients with COPD or autoimmune disorders who require long-term corticosteroid therapy (Kousha et al., 2011). The lower prevalence of corticosteroid use in the co-infection group instead suggests that alternative risk factors may be sufficient to drive *Mucorales* invasion in these patients, including bacterial sepsis-induced immunoparalysis, diabetic ketoacidosis, and disruption of mucosal and epithelial immune barriers (Hotchkiss et al., 2013; van der Poll et al., 2021). Indeed, the high burden of both Gram-positive and Gram-negative bacterial co-infection, together with the profound NK cell depletion observed in this cohort, suggests that acute, multifactorial breakdown of host defences — rather than chronic iatrogenic immunosuppression — may play a more prominent role in the development of *Aspergillus*–*Mucorales* co-infection in this population.

Another layer of complexity is added by the role of Gram-positive bacterial co-infection, which was identified as an associated predictor with borderline significance in the multivariable model (p = 0.061) but consistent importance across all three algorithms. While viral–fungal superinfections have been extensively studied (Verweij et al., 2020; Koehler et al., 2021), bacterial–fungal synergy remains underappreciated. Gram-positive pathogens may facilitate *Mucorales* invasion via epithelial barrier disruption (Peleg et al., 2010; Kruger et al., 2019) and mixed-species biofilm formation (Carolus et al., 2019). Consequently, clinicians should maintain heightened suspicion for occult mucormycosis in patients with invasive aspergillosis who present with concurrent Gram-positive bacterial co-infection and a refractory response to antifungal therapy.

The hyperinflammatory phenotype observed in the co-infection group further elucidates the destructive nature of this syndrome. Our model incorporated CRP as a pivotal surrogate marker of systemic inflammatory burden. The significantly elevated CRP levels observed in the co-infection group align with the distinct pathophysiology of *Mucorales*. Unlike the relatively circumscribed inflammation often seen in IPA, *Mucorales* exhibit aggressive angioinvasion, leading to extensive vascular thrombosis and rapid tissue necrosis (Ibrahim et al., 2012; B. H. S. et al., 2024). This massive tissue infarction precipitates a systemic inflammatory response that far exceeds that of sole *Aspergillus* infection. This angioinvasive phenotype also explains the higher rate of extrapulmonary dissemination (16.7% vs. 0%), underscoring the need for comprehensive distant-organ screening. The more severe anaemia in the mixed group (p = 0.041) likely reflects inflammation-driven suppression of erythropoiesis (Weiss and Goodnough, 2005; Ganz, 2019).

Our study also provides important clinical implications regarding the diagnostic and therapeutic time window. Survival analysis confirmed that initiation of amphotericin B within 7 days of time zero was strongly associated with improved survival. Given that *Mucorales* species are intrinsically resistant to voriconazole (Trifilio et al., 2007; Pongas et al., 2009), failure to recognise co-infection in a timely manner inevitably leads to inadequate antifungal coverage and missed optimal treatment opportunities. However, conventional culture detected *Mucorales* in only 3 of 18 co-infection cases (16.7%), whereas mNGS identified *Mucorales* sequences in 16 cases (88.9%), supporting its prioritisation in the diagnostic workup of critically ill patients with suspected co-infection (Miao et al., 2018; Chen et al., 2021). Taken together, these findings reinforce the importance of early suspicion, rapid molecular diagnostics, and prompt initiation of *Mucorales*-active therapy in this population (Chamilos et al., 2008).

## 5 Limitations

This study has several limitations. First, the single-centre retrospective design and modest sample size of the co-infection group (n = 18) — though reflective of the disease’s rarity — constrain statistical power and external generalisability. The effective events-per-variable ratio (4.5) is below the conventional threshold, and our findings should be interpreted as hypothesis-generating pending external validation (Riley et al., 2019; Collins et al., 2024). Second, diagnostic ascertainment bias may exist due to the evolving availability of mNGS during the study period and the inability to blind the MDT to molecular results in a retrospective setting. Third, the Kaplan–Meier survival analysis was not the primary objective of this study but served to underscore the clinical value of early identification; as an exploratory analysis with a fixed baseline grouping, its results should be interpreted with caution.

## 6 Conclusion

In conclusion, *Aspergillus*–*Mucorales* co-infection in the ICU is associated with high mortality and may present with features of severe immune suppression, heightened systemic inflammation, and a high burden of bacterial co-infection. Based on these observations, the nomogram integrating NK cell count, CRP, corticosteroid use history, and Gram-positive bacterial co-infection demonstrated robust predictive performance and may facilitate the early identification of high-risk patients, guiding timely initiation of *Mucorales*-active therapy to improve prognosis.

## Data Availability

The original contributions presented in the study are included in the article/**Supplementary Material**. Further inquiries can be directed to the corresponding authors.

## Supplementary material

Supplementary Table 1 Univariable logistic regression analysis of all 50 candidate variables.

Supplementary Figure 1 Three-stage feature selection and model development pipeline. The figure illustrates the stepwise variable selection strategy and final model construction process. Stage 1 defined the full candidate pool of 50 clinical, laboratory, and immunological variables from the study cohort (n = 93). Stage 2 applied univariable logistic regression to pre-screen variables, retaining 11 candidates meeting the threshold of p < 0.1 (excluding 39 variables with p ≥ 0.1). Stage 3 employed three complementary machine learning algorithms—LASSO regression (L1 regularization), Ridge regression (L2 regularization), and Random Forest (out-of-bag importance estimation)—to rank the relative importance of the 11 candidate variables independently. Stage 4 applied a dual-criterion selection rule, retaining variables that demonstrated consistent high importance across all three algorithms and possessed clear clinical applicability and interpretability in the ICU setting. Stage 5 presents the four variables selected for the final multivariable logistic regression model and nomogram: NK cell count (negative predictor, OR = 0.000, p = 0.039), corticosteroid use history (negative predictor, OR = 0.161, p = 0.008), C-reactive protein (positive predictor, OR = 1.008, p = 0.024), and Gram-positive bacterial co-infection (positive predictor, OR = 5.597, p = 0.061). The composite model achieved an AUC of 0.878 (95% CI: 0.789–0.967), a Nagelkerke R^2^ of 0.480, and a Hosmer-Lemeshow goodness-of-fit p = 0.849, with an events-per-variable ratio of 4.5. Internal validation by 10-repetition 5-fold cross-validation yielded a mean AUC of 0.860 (± 0.111), confirming acceptable model stability.*Although Gram-positive bacterial co-infection did not reach conventional statistical significance in the multivariable model (p = 0.061), it demonstrated a strong trend (borderline significance) with an OR of 5.597, and was retained in the final model based on its established clinical relevance and consistent high-importance ranking across all three machine learning algorithms (univariable OR = 5.636, p = 0.028). AUC, area under the receiver operating characteristic curve; CI, confidence interval; EPV, events per variable; LASSO, least absolute shrinkage and selection operator; OOB, out-of-bag; OR, odds ratio.
