# Supplementary material for: Confronting the “lethal duo” in the ICU: early identification of Aspergillus–Mucorales co-infection using a clinical-immuno-inflammatory signature
Source: Front Cell Infect Microbiol. 2026 Apr 22;16:1779186. doi: 10.3389/fcimb.2026.1779186 (PMC13144045; doi:10.3389/fcimb.2026.1779186)
Supplement: Supplementary Table 1 — Univariable logistic regression analysis of all 50 candidate variables. [file Table1.docx]

**Supplementary Table S1. Univariable logistic regression analysis of all 50 candidate variables.**

| **Variable** | **OR** | **95% CI** | **p value** |
| --- | --- | --- | --- |
| **SOFA score** | 1.061 | 0.961 – 1.172 | 0.235 |
| APACHE II score | 1.006 | 0.949 – 1.065 | 0.833 |
| Symptom onset to admission (days) | 1.012 | 0.903 – 1.117 | 0.813 |
| Symptom onset to diagnosis (days) | 1.038 | 0.994 – 1.094 | 0.105 |
| Length of hospital stay (days) | 1.003 | 0.986 – 1.018 | 0.727 |
| White blood cell count (×10⁹/L) | 1.031 | 0.974 – 1.088 | 0.274 |
| Neutrophil count (×10⁹/L) | 1.026 | 0.970 – 1.082 | 0.340 |
| **C-reactive protein (mg/L)** | 1.009 | 1.003 – 1.015 | **0.003** |
| Albumin (g/L) | 0.989 | 0.886 – 1.100 | 0.842 |
| D-dimer (mg/L) | 1.039 | 0.985 – 1.094 | 0.138 |
| Total bilirubin (μmol/L) | 0.998 | 0.990 – 1.004 | 0.631 |
| Total protein (g/L) | 0.988 | 0.932 – 1.045 | 0.662 |
| Galactomannan (ODI) | 1.034 | 0.803 – 1.285 | 0.777 |
| Interleukin-6 (pg/mL) | 1.000 | 1.000 – 1.000 | 0.383 |
| Direct bilirubin (μmol/L) | 0.997 | 0.983 – 1.006 | 0.564 |
| RBC (×10¹²/L) | 0.596 | 0.314 – 1.070 | 0.094 |
| Fibrinogen (g/L) | 1.356 | 1.035 – 1.806 | **0.030** |
| Creatinine (μmol/L) | 1.002 | 0.997 – 1.007 | 0.321 |
| Platelet count (×10⁹/L) | 0.999 | 0.993 – 1.003 | 0.576 |
| Hemoglobin (g/L) | 0.980 | 0.960 – 0.999 | **0.046** |
| Procalcitonin (ng/mL) | 1.007 | 0.998 – 1.020 | 0.158 |
| B cells (×10⁹/L) | 0.009 | 0.000 – 0.470 | 0.079 |
| CD3+CD4+ T cells (×10⁹/L) | 0.026 | 0.000 – 0.879 | 0.084 |
| CD3+CD8+ T cells (×10⁹/L) | 0.218 | 0.005 – 2.308 | 0.354 |
| CD3+ T cells (×10⁹/L) | 0.201 | 0.020 – 1.154 | 0.135 |
| **NK cells (×10⁹/L)** | 0.000 | 0.000 – 0.005 | **0.010** |
| eGFR (mL/min/1.73m²) | 0.999 | 0.993 – 1.004 | 0.765 |
| Total lymphocytes (×10⁹/L) | 0.145 | 0.027 – 0.569 | **0.013** |
| (1,3)-β-D-glucan (pg/mL) | 0.999 | 0.997 – 1.001 | 0.465 |
| Male sex | 1.169 | 0.414 – 3.490 | 0.771 |
| Hypertension | 0.903 | 0.302 – 2.556 | 0.850 |
| Coronary heart disease | 1.047 | 0.149 – 4.691 | 0.956 |
| Diabetes mellitus | 1.583 | 0.492 – 4.723 | 0.419 |
| Liver cirrhosis | 0.382 | 0.020 – 2.211 | 0.375 |
| Malignancy | 2.776 | 0.656 – 10.556 | 0.140 |
| Solid organ transplantation | 0.824 | 0.042 – 5.562 | 0.863 |
| Autoimmune disease | 0.737 | 0.157 – 2.589 | 0.660 |
| **Corticosteroid use** | 0.216 | 0.064 – 0.640 | **0.008** |
| Respiratory failure ᵇ | — | — | 0.115 |
| Septic shock | 1.969 | 0.632 – 7.483 | 0.271 |
| Acute kidney injury | 1.300 | 0.436 – 4.420 | 0.651 |
| Gastrointestinal dysfunction | 2.000 | 0.699 – 5.748 | 0.192 |
| Liver dysfunction | 2.353 | 0.694 – 10.847 | 0.208 |
| Mechanical ventilation | 4.983 | 0.915 – 92.978 | 0.132 |
| CRRT | 1.125 | 0.389 – 3.542 | 0.832 |
| ECMO | 4.000 | 0.895 – 17.051 | 0.058 |
| **Gram-positive bacterial co-infection** | 5.636 | 1.462 – 37.283 | **0.028** |
| Gram-negative bacterial co-infection | 2.000 | 0.493 – 13.519 | 0.388 |
| Viral co-infection | 0.885 | 0.303 – 2.804 | 0.826 |
| Extrapulmonary infection ᵇ | — | — | **<0.001** |
| A total of 50 clinical, laboratory, and immunological variables were screened. Variables meeting the pre-screening threshold (p < 0.1) are shaded in yellow (n = 11) and entered the subsequent machine learning importance ranking stage. Variables selected for the final multivariable model are shaded in orange and shown in bold (n = 4): NK cell count, C-reactive protein, corticosteroid use, and Gram-positive bacterial co-infection. | | | |
| ᵃ Respiratory failure and ^b^extrapulmonary infection exhibited complete separation in logistic regression; OR and p values for these two variables were estimated by Fisher's exact test and are reported in Table 1 of the main text. These variables were excluded from subsequent model development owing to unstable coefficient estimation. | | | |
| Abbreviations: OR, odds ratio; CI, confidence interval; SOFA, Sequential Organ Failure Assessment; APACHE II, Acute Physiology and Chronic Health Evaluation II; NK, natural killer; ECMO, extracorporeal membrane oxygenation; CRRT, continuous renal replacement therapy; eGFR, estimated glomerular filtration rate; RBC, red blood cell count. | | | |
