# Supplementary figures and images for: Confronting the “lethal duo” in the ICU: early identification of Aspergillus–Mucorales co-infection using a clinical-immuno-inflammatory signature
Source: Front Cell Infect Microbiol. 2026 Apr 22;16:1779186. doi: 10.3389/fcimb.2026.1779186 (PMC13144045; doi:10.3389/fcimb.2026.1779186)

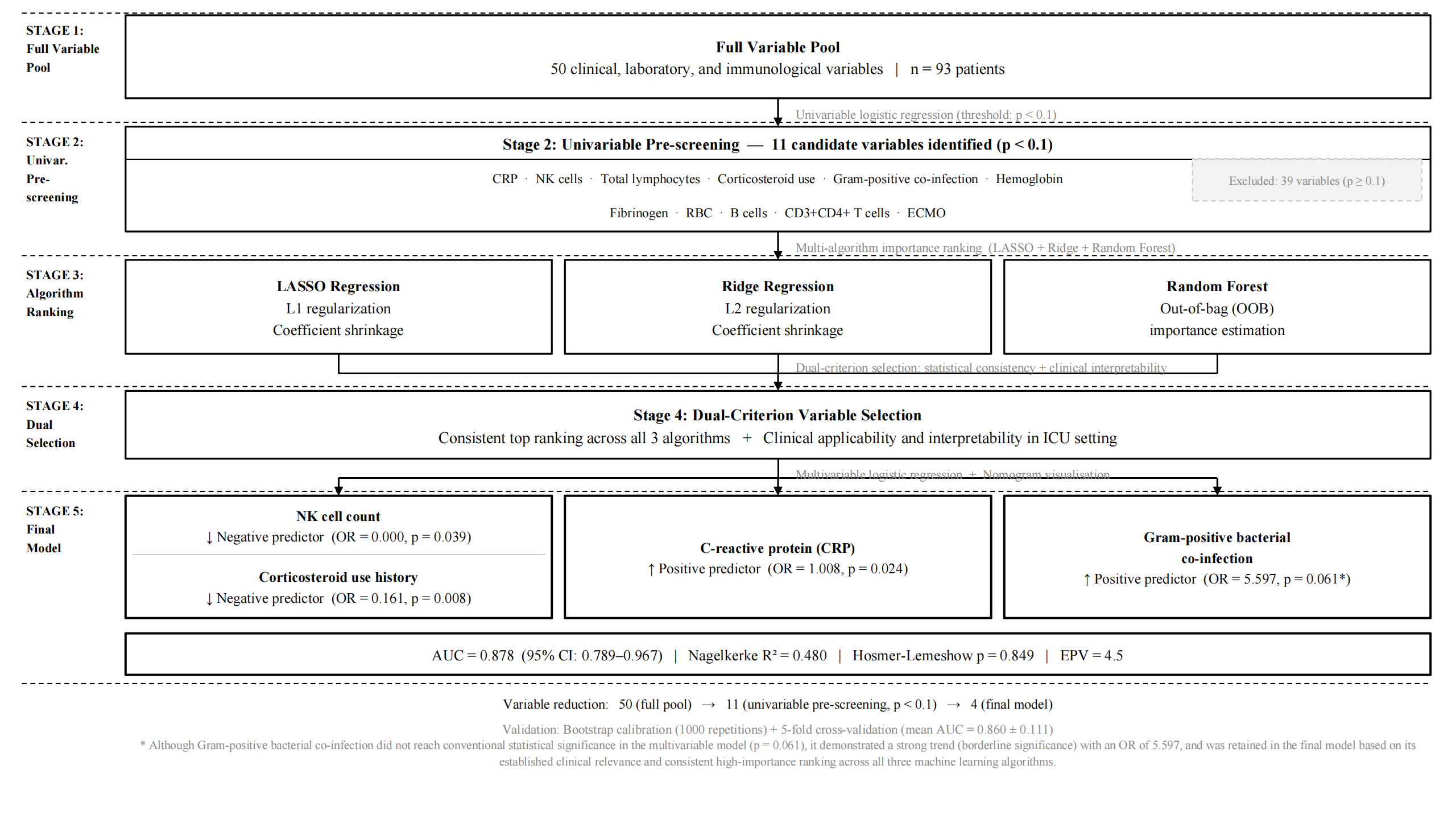

Supplement: Supplementary Figure 1 — Three-stage feature selection and model development pipeline. The figure illustrates the stepwise variable selection strategy and final model construction process. Stage 1 defined the full candidate pool of 50 clinical, laboratory, and immunological variables from the study cohort (n = 93). Stage 2 applied univariable logistic regression to pre-screen variables, retaining 11 candidates meeting the threshold of p < 0.1 (excluding 39 variables with p ≥ 0.1). Stage 3 employed three complementary machine learning algorithms—LASSO regression (L1 regularization), Ridge regression (L2 regularization), and Random Forest (out-of-bag importance estimation)—to rank the relative importance of the 11 candidate variables independently. Stage 4 applied a dual-criterion selection rule, retaining variables that demonstrated consistent high importance across all three algorithms and possessed clear clinical applicability and interpretability in the ICU setting. Stage 5 presents the four variables selected for the final multivariable logistic regression model and nomogram: NK cell count (negative predictor, OR = 0.000, p = 0.039), corticosteroid use history (negative predictor, OR = 0.161, p = 0.008), C-reactive protein (positive predictor, OR = 1.008, p = 0.024), and Gram-positive bacterial co-infection (positive predictor, OR = 5.597, p = 0.061). The composite model achieved an AUC of 0.878 (95% CI: 0.789–0.967), a Nagelkerke R2 of 0.480, and a Hosmer-Lemeshow goodness-of-fit p = 0.849, with an events-per-variable ratio of 4.5. Internal validation by 10-repetition 5-fold cross-validation yielded a mean AUC of 0.860 (± 0.111), confirming acceptable model stability.*Although Gram-positive bacterial co-infection did not reach conventional statistical significance in the multivariable model (p = 0.061), it demonstrated a strong trend (borderline significance) with an OR of 5.597, and was retained in the final model based on its established clinical relevance and consistent high-importan [file Image1.tif]
